# 2-[2-(3-Chloro­phen­yl)-2-oxoeth­yl]-1,2-benzisothia­zol-3(2*H*)-one 1,1-dioxide

**DOI:** 10.1107/S1600536810005428

**Published:** 2010-02-13

**Authors:** Zunera Khalid, Hamid Latif Siddiqui, Matloob Ahmad, Iftikhar Hussain Bukhari, Masood Parvez

**Affiliations:** a Institute of Chemistry, University of the Punjab, Lahore, Pakistan; b Department of Chemistry, University of Sargodha, Sargodha 10400, Pakistan; c Department of Chemistry, The University of Calgary, 2500 University Drive NW, Calgary, Alberta, Canada T2N 1N4

## Abstract

In the title compound, C_15_H_10_ClNO_4_S, the benzothia­zole ring system is essentially planar [maximum deviation = 0.0382 (13) Å for the N atom] and forms a dihedral angle of 74.43 (6)° with the chloro-substituted benzene ring. In the crystal structure, weak inter­molecular C—H⋯O hydrogen bonds form *R*_2_^2^(10) and *R*_2_^2^(16) ring motifs

## Related literature

For the use of 1,2-benzisothia­zoline-3-one 1,1-dioxide (saccharine) as an inter­mediate in the preparation of medicinally important mol­ecules, see: Siddiqui *et al.* (2006 ▶); Zia-ur-Rehman *et al.* (2005 ▶, 2009 ▶). For the biological activity of saccharine, see: Singh *et al.* (2007 ▶); Vaccarino *et al.* (2007 ▶); Kapui *et al.* (2003 ▶). For related structures, see: Ahmad *et al.* (2008 ▶, 2009 ▶). For hydrogen-bonding motifs, see: Bernstein *et al.* (1995 ▶). Zia-ur-Rehman, Choudary & Ahmad (2005 ▶).
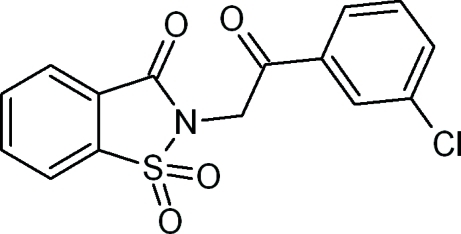


## Experimental

### 

#### Crystal data

C_15_H_10_ClNO_4_S
*M* *_r_* = 335.75
Triclinic, 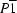

*a* = 7.7258 (4) Å
*b* = 9.0780 (4) Å
*c* = 10.0809 (5) Å
α = 83.884 (3)°
β = 85.092 (3)°
γ = 87.765 (3)°
*V* = 700.10 (6) Å^3^
*Z* = 2
Mo *K*α radiation
μ = 0.44 mm^−1^
*T* = 173 K
0.20 × 0.12 × 0.10 mm

#### Data collection

Nonius diffractometer with Bruker APEXII CCD
Absorption correction: multi-scan (*SORTAV*; Blessing, 1997 ▶) *T*_min_ = 0.917, *T*_max_ = 0.957
5768 measured reflections
3157 independent reflections
2881 reflections with (*I*) > 2.0 σ(*I*)
*R*_int_ = 0.025

#### Refinement

*R*[*F* ^2^ > 2σ(*F* ^2^)] = 0.045
*wR*(*F* ^2^) = 0.130
*S* = 1.06
3157 reflections
199 parameters
H-atom parameters constrained
Δρ_max_ = 0.36 e Å^−3^
Δρ_min_ = −0.40 e Å^−3^

### 

Data collection: *COLLECT* (Hooft, 1998 ▶); cell refinement: *HKL* *DENZO* (Otwinowski & Minor, 1997 ▶); data reduction: *SCALEPACK* (Otwinowski & Minor, 1997 ▶); program(s) used to solve structure: *SHELXS97* (Sheldrick, 2008 ▶); program(s) used to refine structure: *SHELXL97* (Sheldrick, 2008 ▶); molecular graphics: *ORTEP-3 for Windows* (Farrugia, 1997 ▶); software used to prepare material for publication: *SHELXL97*.

## Supplementary Material

Crystal structure: contains datablocks Global, I. DOI: 10.1107/S1600536810005428/lh2992sup1.cif

Structure factors: contains datablocks I. DOI: 10.1107/S1600536810005428/lh2992Isup2.hkl

Additional supplementary materials:  crystallographic information; 3D view; checkCIF report

## Figures and Tables

**Table 1:** Hydrogen-bond geometry (Å, °)

*D*—H⋯*A*	*D*—H	H⋯*A*	*D*⋯*A*	*D*—H⋯*A*
C2—H2⋯O2^i^	0.95	2.40	3.249 (3)	148
C8—H8*A*⋯O1^ii^	0.99	2.45	3.378 (3)	156
C11—H11⋯O3^iii^	0.95	2.44	3.382 (3)	173

Symmetry codes: (i) 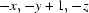; (ii) 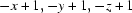; (iii) 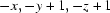.

**Fig. 1.**
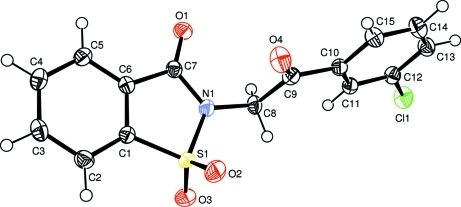
ORTEP-3 (Farrugia, 1997) drawing of (I) with displacement ellipsoids plotted at 50% probability level.

**Fig. 2.**
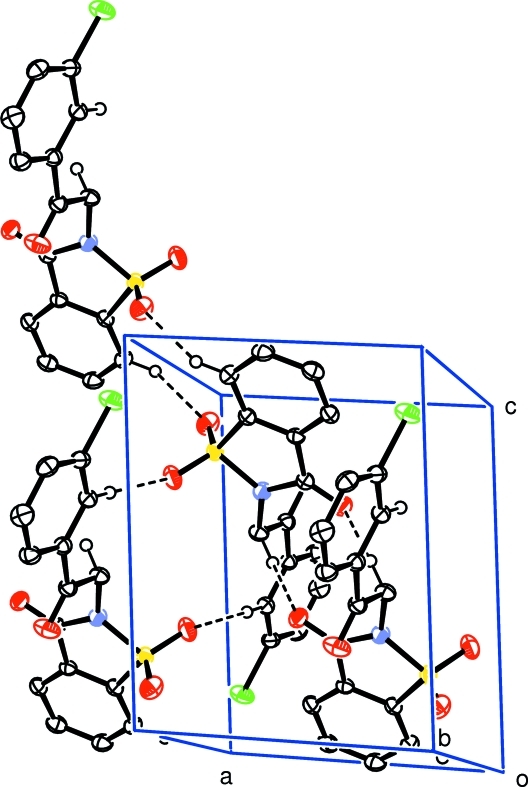
Unit cell packing of (I) showing non-classical hydrogen bonding interaction with dashed lines; H-atoms not involved in H-bonds have been excluded for clarity.

## supplementary crystallographic information

### Comment

1,2-Benzisothiazoline-3-one 1,1-dioxide (saccharine) is an important starting material for the synthesis of different heterocyclic compounds and plays a role as an intermediate for the preparation of medicinally important molecules (Siddiqui *et al.*, 2006; Zia-ur-Rehman *et al.*, 2005; Zia-ur-Rehman *et al.*, 2009). Various derivatives of saccharin are known to be cyclooxygenase-2 (COX-2) inhibitors (Singh *et al.*, 2007), analgesic (Vaccarino *et al.*, 2007), human leucocyte elastase (HLE) inhibitors (Kapui *et al.*, 2003) etc. In continuation of our research on the synthesis of potential biologically active derivatives of benzothiazines (Ahmad *et al.*, 2008; Ahmad *et al.*, 2009), we herein report the crystal structure of the title compound (I).

The molecular structure of the title compund is shown in (Fig. 1). The benzothiazole moiety (S1/N1/C1—C7) is essentially planar (maximum deviation = 0.0382 (13) Å for atom N1) and lies at an angle 74.43 (6) ° with respect to the C10—C15 benzene ring. The structure is devoid of any classical hydrogen bonds. However, non-classical hydrogen bonding interactions of the type C—H···O are present in the crystal structure resulting in ten and sixteen membered macrocyclic rings in R_2_^2^(10) and R_2_^2^(16) motifs (Bernstein *et al.*, 1995) (Fig. 2 and Table 1).

### Experimental

3-Chlorophenacyl bromide (5.60 g, 0.024 mol) was slowly added to a suspension of sodium saccharine (5 g, 0.024 mol) in dimethylformamide (15 ml) and the mixture was stirred at 383 K for 3 hours under anhydrous conditions. On completion of reaction (indicated by TLC), the mixture was poured on crushed ice and the precipitates formed were filtered and washed with excess of distilled water and cold ethanol respectively. The crystals of the title compound suitable for XRD were grown from a solution of chloroform-methanol (3:1).

### Refinement

All H-atoms were located from the difference Fourier maps and were included in the refinements at geometrically idealized positions with C—H distances = 0.95 and 0.99 Å for aryl and methylene H-atoms, respectively, and U_iso_ = 1.2 times U_eq_ of the C-atoms to which they were bonded. The final difference map was free of chemically significant features.

### Crystal data

**Table:**

C_15_H_10_ClNO_4_S	*Z* = 2
*M**_r_* = 335.75	*F*(000) = 344
Triclinic, *P*1	*D*_x_ = 1.593 Mg m^−^^3^
Hall symbol: -P 1	Melting point: 488 K
*a* = 7.7258 (4) Å	Mo *K*α radiation, λ = 0.71073 Å
*b* = 9.0780 (4) Å	Cell parameters from 2955 reflections
*c* = 10.0809 (5) Å	θ = 1.0–27.5°
α = 83.884 (3)°	µ = 0.44 mm^−^^1^
β = 85.092 (3)°	*T* = 173 K
γ = 87.765 (3)°	Prism, white
*V* = 700.10 (6) Å^3^	0.20 × 0.12 × 0.10 mm

### Data collection

**Table:**

Nonius APEXII CCD diffractometer	3157 independent reflections
Radiation source: fine-focus sealed tube	2881 reflections with (*I*) > 2.0 σ(*I*)
graphite	*R*_int_ = 0.025
φ & ω scans	θ_max_ = 27.5°, θ_min_ = 2.3°
Absorption correction: multi-scan (*SORTAV*; Blessing, 1997)	*h* = −10→9
*T*_min_ = 0.917, *T*_max_ = 0.957	*k* = −11→11
5768 measured reflections	*l* = −12→13

### Refinement

**Table:**

Refinement on *F*^2^	Primary atom site location: structure-invariant direct methods
Least-squares matrix: full	Secondary atom site location: difference Fourier map
*R*[*F*^2^ > 2σ(*F*^2^)] = 0.045	Hydrogen site location: inferred from neighbouring sites
*wR*(*F*^2^) = 0.130	H-atom parameters constrained
*S* = 1.06	*w* = 1/[σ^2^(*F*_o_^2^) + (0.0738*P*)^2^ + 0.5259*P*] where *P* = (*F*_o_^2^ + 2*F*_c_^2^)/3
3157 reflections	(Δ/σ)_max_ < 0.001
199 parameters	Δρ_max_ = 0.36 e Å^−^^3^
0 restraints	Δρ_min_ = −0.40 e Å^−^^3^

### Special details

**Table:**

Experimental. IR (KBr): 1737, 1690, 1341, 1151 cm^-1^, ^1^H NMR: (DMSO-d_6_) δ: 5.40 (s, 2H, CH_2_), 7.43 (dd, 1H, J1 = 2.4 Hz, J2 = 8.4 Hz, Ar—H), 7.50 (t, 1H, J = 8.0 Hz, Ar—H), 7.58 (t, 1H, J = 2.4 Hz, Ar—H), 7.65 (d, 1H, J = 7.6 Hz, Ar—H), 8.05 (t, 1H, J = 7.6 Hz, Ar—H), 8.11 (t, 1H, J = 7.6 Hz, Ar—H), 8.17 (d, 1H, J = 7.6 Hz, Ar—H), 8.23 (d, 1H, J = 7.2 Hz, Ar—H). MS m/z: 335.8[M^+^].
Geometry. All esds (except the esd in the dihedral angle between two l.s. planes) are estimated using the full covariance matrix. The cell esds are taken into account individually in the estimation of esds in distances, angles and torsion angles; correlations between esds in cell parameters are only used when they are defined by crystal symmetry. An approximate (isotropic) treatment of cell esds is used for estimating esds involving l.s. planes.
Refinement. Refinement of *F*^2^ against ALL reflections. The weighted *R*-factor wR and goodness of fit *S* are based on *F*^2^, conventional *R*-factors *R* are based on *F*, with *F* set to zero for negative *F*^2^. The threshold expression of *F*^2^ > σ(*F*^2^) is used only for calculating *R*-factors(gt) etc. and is not relevant to the choice of reflections for refinement. *R*-factors based on *F*^2^ are statistically about twice as large as those based on *F*, and *R*- factors based on ALL data will be even larger.

### Fractional atomic coordinates and isotropic or equivalent isotropic displacement parameters (Å^2^)

**Table:**

	*x*	*y*	*z*	*U*_iso_*/*U*_eq_	
Cl1	0.05636 (8)	0.97635 (7)	0.83644 (5)	0.03594 (18)	
S1	0.13366 (6)	0.50633 (5)	0.22038 (5)	0.02250 (15)	
O1	0.57018 (19)	0.54728 (17)	0.34507 (15)	0.0275 (3)	
O2	0.0709 (2)	0.62625 (18)	0.13199 (17)	0.0340 (4)	
O3	0.0067 (2)	0.42357 (19)	0.30583 (17)	0.0341 (4)	
O4	0.3503 (2)	0.85538 (18)	0.25316 (16)	0.0360 (4)	
N1	0.2798 (2)	0.56575 (19)	0.31383 (17)	0.0237 (4)	
C1	0.2881 (2)	0.3923 (2)	0.13935 (19)	0.0206 (4)	
C2	0.2593 (3)	0.2980 (2)	0.0452 (2)	0.0259 (4)	
H2	0.1469	0.2874	0.0166	0.031*	
C3	0.4039 (3)	0.2192 (2)	−0.0055 (2)	0.0285 (4)	
H3	0.3898	0.1525	−0.0700	0.034*	
C4	0.5687 (3)	0.2356 (2)	0.0359 (2)	0.0279 (4)	
H4	0.6648	0.1808	−0.0012	0.034*	
C5	0.5948 (3)	0.3314 (2)	0.1312 (2)	0.0239 (4)	
H5	0.7071	0.3424	0.1598	0.029*	
C6	0.4518 (3)	0.4102 (2)	0.18287 (18)	0.0197 (4)	
C7	0.4493 (3)	0.5143 (2)	0.28697 (19)	0.0207 (4)	
C8	0.2318 (3)	0.6673 (2)	0.4143 (2)	0.0237 (4)	
H8A	0.2891	0.6334	0.4968	0.028*	
H8B	0.1046	0.6660	0.4367	0.028*	
C9	0.2845 (3)	0.8255 (2)	0.3652 (2)	0.0232 (4)	
C10	0.2549 (3)	0.9392 (2)	0.46202 (19)	0.0216 (4)	
C11	0.1755 (3)	0.9062 (2)	0.5907 (2)	0.0219 (4)	
H11	0.1351	0.8095	0.6193	0.026*	
C12	0.1567 (3)	1.0164 (2)	0.6761 (2)	0.0237 (4)	
C13	0.2149 (3)	1.1584 (2)	0.6376 (2)	0.0277 (4)	
H13	0.2020	1.2324	0.6979	0.033*	
C14	0.2926 (3)	1.1900 (2)	0.5085 (2)	0.0307 (5)	
H14	0.3325	1.2868	0.4804	0.037*	
C15	0.3124 (3)	1.0828 (2)	0.4209 (2)	0.0266 (4)	
H15	0.3648	1.1062	0.3328	0.032*	

### Atomic displacement parameters (Å^2^)

**Table:**

	*U*^11^	*U*^22^	*U*^33^	*U*^12^	*U*^13^	*U*^23^
Cl1	0.0441 (3)	0.0384 (3)	0.0246 (3)	−0.0009 (2)	0.0080 (2)	−0.0084 (2)
S1	0.0185 (2)	0.0232 (3)	0.0262 (3)	0.00100 (18)	−0.00166 (18)	−0.00494 (19)
O1	0.0254 (7)	0.0312 (8)	0.0276 (8)	−0.0025 (6)	−0.0057 (6)	−0.0074 (6)
O2	0.0318 (8)	0.0319 (8)	0.0386 (9)	0.0082 (7)	−0.0110 (7)	−0.0020 (7)
O3	0.0245 (8)	0.0375 (9)	0.0398 (9)	−0.0060 (7)	0.0071 (7)	−0.0073 (7)
O4	0.0505 (10)	0.0325 (8)	0.0234 (8)	−0.0037 (7)	0.0081 (7)	−0.0037 (6)
N1	0.0221 (8)	0.0248 (8)	0.0252 (8)	0.0024 (7)	−0.0022 (6)	−0.0087 (7)
C1	0.0206 (9)	0.0200 (9)	0.0207 (9)	0.0005 (7)	0.0003 (7)	−0.0017 (7)
C2	0.0257 (10)	0.0271 (10)	0.0258 (10)	−0.0028 (8)	−0.0042 (8)	−0.0047 (8)
C3	0.0366 (12)	0.0261 (10)	0.0238 (10)	−0.0025 (8)	−0.0007 (8)	−0.0083 (8)
C4	0.0300 (10)	0.0279 (10)	0.0250 (10)	0.0041 (8)	0.0027 (8)	−0.0047 (8)
C5	0.0218 (9)	0.0254 (10)	0.0238 (10)	0.0007 (8)	0.0009 (7)	−0.0016 (8)
C6	0.0234 (9)	0.0185 (9)	0.0168 (8)	−0.0013 (7)	−0.0005 (7)	−0.0007 (7)
C7	0.0224 (9)	0.0182 (9)	0.0210 (9)	−0.0007 (7)	−0.0010 (7)	−0.0007 (7)
C8	0.0280 (10)	0.0216 (9)	0.0217 (9)	0.0010 (8)	0.0010 (8)	−0.0067 (7)
C9	0.0219 (9)	0.0258 (10)	0.0216 (9)	0.0011 (7)	−0.0014 (7)	−0.0025 (8)
C10	0.0217 (9)	0.0220 (9)	0.0215 (9)	0.0002 (7)	−0.0020 (7)	−0.0032 (7)
C11	0.0222 (9)	0.0209 (9)	0.0227 (9)	0.0007 (7)	−0.0007 (7)	−0.0031 (7)
C12	0.0252 (10)	0.0270 (10)	0.0193 (9)	0.0017 (8)	−0.0025 (7)	−0.0041 (7)
C13	0.0317 (11)	0.0244 (10)	0.0289 (11)	0.0010 (8)	−0.0073 (8)	−0.0076 (8)
C14	0.0390 (12)	0.0214 (10)	0.0319 (11)	−0.0038 (9)	−0.0053 (9)	−0.0007 (8)
C15	0.0325 (11)	0.0244 (10)	0.0228 (10)	−0.0032 (8)	−0.0024 (8)	−0.0003 (8)

### Geometric parameters (Å, °)

**Table:**

Cl1—C12	1.740 (2)	C5—C6	1.387 (3)
S1—O2	1.4285 (16)	C5—H5	0.9500
S1—O3	1.4297 (16)	C6—C7	1.483 (3)
S1—N1	1.6707 (18)	C8—C9	1.528 (3)
S1—C1	1.755 (2)	C8—H8A	0.9900
O1—C7	1.207 (2)	C8—H8B	0.9900
O4—C9	1.206 (3)	C9—C10	1.493 (3)
N1—C7	1.388 (3)	C10—C11	1.395 (3)
N1—C8	1.456 (2)	C10—C15	1.403 (3)
C1—C2	1.381 (3)	C11—C12	1.384 (3)
C1—C6	1.394 (3)	C11—H11	0.9500
C2—C3	1.393 (3)	C12—C13	1.387 (3)
C2—H2	0.9500	C13—C14	1.391 (3)
C3—C4	1.392 (3)	C13—H13	0.9500
C3—H3	0.9500	C14—C15	1.377 (3)
C4—C5	1.394 (3)	C14—H14	0.9500
C4—H4	0.9500	C15—H15	0.9500
			
O2—S1—O3	116.98 (10)	N1—C7—C6	108.78 (17)
O2—S1—N1	110.34 (9)	N1—C8—C9	111.68 (16)
O3—S1—N1	108.98 (10)	N1—C8—H8A	109.3
O2—S1—C1	112.54 (10)	C9—C8—H8A	109.3
O3—S1—C1	112.63 (10)	N1—C8—H8B	109.3
N1—S1—C1	92.62 (9)	C9—C8—H8B	109.3
C7—N1—C8	122.70 (17)	H8A—C8—H8B	107.9
C7—N1—S1	115.44 (14)	O4—C9—C10	121.91 (19)
C8—N1—S1	121.85 (14)	O4—C9—C8	120.72 (19)
C2—C1—C6	122.96 (18)	C10—C9—C8	117.36 (17)
C2—C1—S1	127.13 (16)	C11—C10—C15	119.82 (19)
C6—C1—S1	109.90 (14)	C11—C10—C9	122.23 (18)
C1—C2—C3	116.44 (19)	C15—C10—C9	117.94 (18)
C1—C2—H2	121.8	C12—C11—C10	118.92 (18)
C3—C2—H2	121.8	C12—C11—H11	120.5
C4—C3—C2	121.64 (19)	C10—C11—H11	120.5
C4—C3—H3	119.2	C11—C12—C13	121.94 (19)
C2—C3—H3	119.2	C11—C12—Cl1	119.16 (16)
C3—C4—C5	120.9 (2)	C13—C12—Cl1	118.90 (16)
C3—C4—H4	119.5	C12—C13—C14	118.45 (19)
C5—C4—H4	119.5	C12—C13—H13	120.8
C6—C5—C4	118.02 (19)	C14—C13—H13	120.8
C6—C5—H5	121.0	C15—C14—C13	121.0 (2)
C4—C5—H5	121.0	C15—C14—H14	119.5
C5—C6—C1	119.99 (18)	C13—C14—H14	119.5
C5—C6—C7	126.87 (18)	C14—C15—C10	119.9 (2)
C1—C6—C7	113.12 (17)	C14—C15—H15	120.1
O1—C7—N1	123.66 (18)	C10—C15—H15	120.1
O1—C7—C6	127.50 (18)		
			
O2—S1—N1—C7	−111.61 (16)	C8—N1—C7—C6	178.35 (16)
O3—S1—N1—C7	118.65 (15)	S1—N1—C7—C6	−3.0 (2)
C1—S1—N1—C7	3.62 (16)	C5—C6—C7—O1	−0.6 (3)
O2—S1—N1—C8	67.08 (18)	C1—C6—C7—O1	177.71 (19)
O3—S1—N1—C8	−62.65 (18)	C5—C6—C7—N1	−177.90 (19)
C1—S1—N1—C8	−177.68 (16)	C1—C6—C7—N1	0.5 (2)
O2—S1—C1—C2	−69.4 (2)	C7—N1—C8—C9	75.9 (2)
O3—S1—C1—C2	65.5 (2)	S1—N1—C8—C9	−102.69 (18)
N1—S1—C1—C2	177.32 (19)	N1—C8—C9—O4	3.1 (3)
O2—S1—C1—C6	110.17 (15)	N1—C8—C9—C10	−175.59 (17)
O3—S1—C1—C6	−114.98 (15)	O4—C9—C10—C11	178.6 (2)
N1—S1—C1—C6	−3.15 (15)	C8—C9—C10—C11	−2.8 (3)
C6—C1—C2—C3	0.1 (3)	O4—C9—C10—C15	−2.4 (3)
S1—C1—C2—C3	179.62 (16)	C8—C9—C10—C15	176.20 (18)
C1—C2—C3—C4	−0.4 (3)	C15—C10—C11—C12	−0.7 (3)
C2—C3—C4—C5	0.5 (3)	C9—C10—C11—C12	178.30 (18)
C3—C4—C5—C6	−0.3 (3)	C10—C11—C12—C13	−0.2 (3)
C4—C5—C6—C1	0.0 (3)	C10—C11—C12—Cl1	179.53 (15)
C4—C5—C6—C7	178.24 (18)	C11—C12—C13—C14	0.7 (3)
C2—C1—C6—C5	0.1 (3)	Cl1—C12—C13—C14	−179.03 (17)
S1—C1—C6—C5	−179.49 (15)	C12—C13—C14—C15	−0.3 (3)
C2—C1—C6—C7	−178.41 (18)	C13—C14—C15—C10	−0.5 (3)
S1—C1—C6—C7	2.0 (2)	C11—C10—C15—C14	1.0 (3)
C8—N1—C7—O1	1.0 (3)	C9—C10—C15—C14	−178.0 (2)
S1—N1—C7—O1	179.65 (16)		

### Hydrogen-bond geometry (Å, °)

**Table:**

*D*—H···*A*	*D*—H	H···*A*	*D*···*A*	*D*—H···*A*
C2—H2···O2^i^	0.95	2.40	3.249 (3)	148
C8—H8A···O1^ii^	0.99	2.45	3.378 (3)	156
C11—H11···O3^iii^	0.95	2.44	3.382 (3)	173

Symmetry codes: (i) −*x*, −*y*+1, −*z*; (ii) −*x*+1, −*y*+1, −*z*+1; (iii) −*x*, −*y*+1, −*z*+1.
